# The role of cell free DNA and liquid biopsies in haematological conditions

**DOI:** 10.20517/cdr.2019.93

**Published:** 2020-03-13

**Authors:** Nicholas Bingham, Andrew Spencer

**Affiliations:** Australian Centre for Blood Diseases, Monash University, Victoria 3181, Australia.

**Keywords:** cfDNA, liquid biopsy, haematology, myeloma, lymphoma, leukaemia

## Abstract

Cell free nucleic acids (CFNAs) are nucleic acids released from cells that circulate within bodily fluids. Recent advances in molecular techniques have led the ability to interrogate CFNAs in a clinically meaningful way, for example the identification and assessment of foetal CFNAs in maternal blood, allowing minimally invasive testing for foetal genetic abnormalities. The majority of CFNAs arise from haemopoietic cells, making it a particularly rich source of genetic information in haematological conditions. Furthermore, the innate genetic heterogeneity of haematological malignancies, as epitomised by multiple myeloma, lend itself well to “liquid biopsies”. This approach promises to provide a more wholistic assessment of whole disease genetics, especially when contrasted against the current gold-standard of single site tissue biopsies. This review briefly summarises the definitions and physiology of CFNAs, both cell free DNA (cfDNA) and extracellular RNA (exRNA), before exploring the literature surrounding the current and future roles of cfDNA in the haematological malignancies and patient care.

## Introduction

Cell free nucleic acids (CFNAs) were first identified in the blood in 1948^[1]^, although many decades passed before molecular techniques with adequate sensitivity to usefully interrogate the CFNAs were developed. Given the relative ease with which most body fluids are accessed, the nucleic acids found in blood and other body fluids are becoming an increasingly attractive source of genetic material for many applications. The first cancer-associated mutations identified in CFNAs were in blood taken from patients with myelodysplastic syndrome^[2,3]^ and indeed most cell free DNA (cfDNA) is derived from haemopoietic cells^[4,5]^. Accordingly, the study of CFNAs is tightly interconnected with haematology, and it is in haematological conditions that interrogation of CFNA arguably holds the most promise.

This review briefly summarises the definitions and physiology of CFNAs relevant to liquid biopsies in haematology, with a focus on what is known about cfDNA in haematological conditions and the future potential of liquid biopsies in the clinic.

## Definitions

The nucleic acids found in the plasma exist as either DNA or RNA. cfDNA describes all of the DNA found in the plasma, while circulating tumour DNA (ctDNA) is the portion of cfDNA that is derived solely from tumour cells. cfDNA is comprised of short fragments of double-stranded DNA^[5]^, often wrapped around histone proteins (the subunit known as a nucleosome) sometimes with an additional H1 protein (known as a chromatosome). cfDNA is mainly found free in the plasma as these chromatosomes. Historically, it was considered that a small proportion of cfDNA was found within exosomes, the small membrane-bound vesicles released from cells into body fluids^[6,7]^, although more recent data suggest this is not the case^[8]^. This may relate to differences in the methods of exosome isolation, with resultant differences in exosome purity accounting for this observed variation.

RNA can also be found circulating in the blood as extracellular RNA (exRNA); however, the majority of exRNA is found within exosomes^[9]^. The systematic interrogation of exRNA and thus the identification of clinical applications of exRNA in haematology are significantly less developed than cfDNA, as such this review focuses on the role cfDNA in haematological conditions.

## Physiology of CFNA

CFNAs are released into body fluids from cells dying due to apoptosis or necrosis^[5]^. The release of CFNAs can be spontaneous^[10,11]^ or triggered by chemotherapy- or radiation therapy-induced apoptosis. After cell death, macrophages in the tissue phagocytose cellular debris, but some nucleic acids escape this process and enter the blood stream^[12]^. In addition, it has been recognised that some cells also actively secrete nucleic acids into the body fluids^[6,13]^.

CFNAs are released by tumour cells as well as normal, healthy cells. Moreover, the amount of CFNA found in the blood varies during certain physiological or pathological states. However, it has been long established that the quantity of cfDNA is higher in patients with malignancies^[14]^, averaging 180 ng/mL compared to 30 ng/mL in healthy controls^[15,16]^. The ctDNA released from tumour cells comprises a variable proportion of this increase, with a range of 3%-93%^[11]^. Other non-malignant conditions also increase the levels of cfDNA, with elevated levels of cfDNA, when compared to normal individuals, having been identified in a number of disease states, including allogeneic transplantation^[17]^, autoimmune diseases, trauma and after exercise^[18,19]^. In pregnant women, 10%-15% of cfDNA is of foetal origin and this is now routinely used to detect foetal chromosomal abnormalities^[20]^.

**Figure 1:**
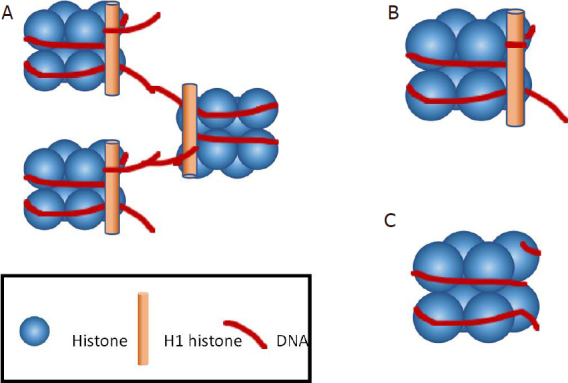
DNA is stored within a cell as chromatin, comprising a single DNA strand wrapped around multiple subunits known as chromatosomes and nucleosomes. A: several subunits are linked by DNA to form chromatin. cfDNA is found as both chromatosomes and nucleosomes; B: a chromatosome is comprised of a core of eight histone proteins, wrapped in DNA of typically 167 base pairs length, with the linkage section of DNA and the H1 histone; C: by contrast, a nucleosome subunit comprises the eight-histone core without the H1 histone protein and loss of the linkage segment of DNA, making the typical length of DNA in the nucleosome only 147 base pairs in length. cfDNA: cell free DNA

cfDNA is released as short fragments of double-stranded DNA, with a typical length of 166-167 base pairs^[5,21]^. Interestingly, ctDNA is usually shorter than cfDNA, with an average length of 132-145 base pairs^[21]^. The typical lengths of ctDNA and cfDNA of 147 and 167 base pairs correlate with the length of DNA associated with a nucleosome and a chromatosome, respectively^[22]^. A total of 147 base pairs represents the length of DNA wrapped around the four histone proteins that comprise a nucleosome, whereas 167 base pairs is the nucleosome DNA with a section of linker DNA attached^[5,22]^ [Figure 1]. Importantly, the association of cfDNA with the nucleosome proteins protects it from cleavage by nucleases^[22]^. Circulating CFNAs are rapidly cleared by the kidneys and liver from the circulation with a half-life measured in minutes to several hours^[15]^, whereas the cfDNA found within exosomes is likely to be protected from this rapid degradation^[6,7]^.

## Analysing cfDNA in haematological conditions

In mature B-cell non-Hodgkin’s lymphomas (NHL) and plasma cell dyscrasias (PCD), malignant clones arise from post-germinal centre B-cells, having undergone rearrangement of the immunoglobulin heavy chain gene as part of the process of somatic hypermutation and immunoglobulin class switching to optimise the affinity of immunoglobulins for specific antigens^[23]^. Theoretically, therefore, all the clonal cells present in either NHL or PCD patients contain identically mutated, patient-specific IgH variable regions.

This clonally rearranged Ig heavy chain DNA was first identified in cfDNA in 1997^[24]^, and now, with the development of next-generation sequencing techniques (NGS), it is possible to detect and quantify the *IgH* gene rearrangements within the cfDNA of both NHL and multiple myeloma (MM) patients^[25]^.

In addition to IgH sequencing, cfDNA can be used to identify disease-specific somatic mutations in haematology patients^[26]^. Once an appropriate driver mutation is identified, subsequent digital PCR can be used to monitor changes in the level of the mutation over time^[26]^ and these changes can be quantitative biomarkers of both disease response and relapse.

An additional utility of cfDNA interrogation is the possibility of examining patterns of DNA methylation. Methylated cfDNA was identified and described in patients with lymphoproliferative disorders in 2003^[27]^. In a subsequent study, *DLC-1* gene methylation in NHL patients was compared between blood and tumour tissue samples and found to be concordant between sites^[28]^. In this context, methylation patterns can be used to monitor disease progress and may also be of prognostic significance, as discussed below.

### Limitations of cfDNA

There are several inherent limitations in the use of cfDNA in haematological conditions. The use of cfDNA may be confounded in some settings due to the presence of coincident clonal haemopoiesis of indeterminant significance (CHIP)^[29]^. CHIP is a condition with an incidence that increases with age, characterised by a population of clonal haemopoietic cells in the circulation. As most cfDNA is derived from the haemopoietic system^[4,5]^, the presence of CHIP will be associated with the presence of cfDNA derived mutations in the circulation that are separate from an underlying, unrelated malignancy. CHIP-associated mutations are well characterised, and thus may be excluded when cfDNA analysis is being performed for non-haematological malignancies. However, when cfDNA is being used to interrogate a possible haematological condition, CHIP mutations may be indistinguishable from mutations sometimes identified in the condition of interest.

Further to this, many haematological conditions lack tumour-specific mutations with identical mutations found across a range of diverse haematological conditions. Additionally, the clonally rearranged Ig heavy chain requires individually tailored primers to identify the mutation, making the process labour-intensive and expensive and requiring the availability of suitable diagnostic tissue for initial characterisation of the specific IgH mutation^[30]^. Similar to the situation found with coincident CHIP, recurrent somatic mutations can be detected but will not be diagnostic of a specific haematological disease, mandating the requirement for ongoing conventional diagnostic testing.

There are also more general technical limitations for the use of cfDNA that impact the application of cfDNA approaches in haematological conditions and more broadly. Accurate genomic information from cfDNA is critically reliant on the isolation of good quality DNA from samples, requiring standardised and validated protocols for gathering samples and extracting cfDNA^[31]^. However, there is still a measurable rate of test failure despite these approaches^[32]^. The interrogation of cfDNA can also be affected by the short length of cfDNA fragments, for example in the library preparation step for sequencing reactions^[33]^. Furthermore, the low relative abundance of mutated alleles in cfDNA also impacts the sensitivity of this approach. As cfDNA contains DNA from all tumours throughout the body, and thus all disease subclones, the relative abundance of the individual mutations present is less. It is further diluted amongst the cfDNA from healthy cells. Thus, cfDNA requires highly sensitive next generation techniques with sufficient read depth or digital PCR to target specific mutations that may be present at lower allelic frequencies^[18,34]^.

Technical limitations aside, the major limitations related to the utilisation of cfDNA applications is a lack of randomised control trials that guide its use in the clinic. While undoubtedly providing genomic information and correlating with outcomes, there is at this time no evidence that altering treatment on the basis of cfDNA findings changes patient outcomes when compared to current clinical practice. It is, however, worth noting that in non-haematological conditions cfDNA is in regular clinical use, including foetal karyotyping in pregnancy and in epidermal growth factor receptor mutation testing in lung cancer^[22,35]^. Ongoing clinical trials now frequently include cfDNA analyses to establish clinical utility, but to date data supporting a role for cfDNA in the haematology clinic are preliminary.

### cfDNA in MM

MM is the archetypal haematological disease for studying the utility of cfDNA and liquid biopsies. The marked spatial and temporal genetic heterogeneity of MM is well established, and the gold standard bone marrow biopsy is now recognised as not being genomically representative of the disease as a whole^[36-38]^. cfDNA, representing DNA from the tumour in its entirety, better captures the genetic diversity of MM compared with bone marrow (BM) samples alone^[35-38]^.

Traditionally, disease monitoring of MM patients involves sequential serum paraprotein estimation, although oligosecretory and non-secretory MM patients require regular bone marrow biopsies or positron emission tomography/computed tomography (PET/CT) scans to monitor disease, which are invasive, costly or involve radiation associated risks. Furthermore, in advanced disease, MM progression does not infrequently manifest as light chain escape, where paraprotein estimations are no longer representative of disease burden, with tumour cells secreting only light chains, or indeed becoming entirely non-secretory^[39]^. Evidently, a non-invasive but representative method of disease burden monitoring that can be used in all MM patients would be highly desirable; cfDNA could potentially fulfil this role. As described below, analysis of cfDNA can provide prognostic as well as genomic information; it can also be used to measure response to treatment, monitor for disease progression and has been correlated with survival.

With regards to prognosis in MM, a higher number of mutations and higher fractional abundance (proportion of mutated versus wild type alleles from a specific locus) of these mutations detected in ctDNA at the start of treatment correlates with worse survival^[38]^. Furthermore, in the same study, response to therapy was predicted by a reduction in the levels of ctDNA at Day 5, highlighting the promise of ctDNA in the early detection of patients failing therapy^[39]^. The possibility of predicting non-responders and an early change of therapy is an attractive theoretical way to improve patient outcomes, although clinical trials are required to confirm this.

In MM, the end-stages of disease are characterised by the development of extramedullary plasmacytomas, where clonal plasma cell tumours arise outside of the BM niche and are typically refractory to chemotherapy^[40]^. cfDNA is of particular interest in this group of patients, given the difficulty in obtaining biopsies, and that different sites frequently yield different genetic abnormalities^[36]^. Targeted amplicon sequencing of cfDNA using a panel of MM-associated genes has been used in conjunction with multiple biopsies and PET-CT scanning in patients with extramedullary (EM) disease, generating informative quantitative and qualitative data^[37]^. Mutations were identified in cfDNA not present in the BM but found at sites of EM disease, indicating cfDNA may be able to characterise EM disease without a biopsy. Furthermore, changes in cfDNA over time correlated with tumour burden, indicating a possible role in monitoring response to therapy. This study also demonstrated clonal evolution in the cfDNA of a MM patient over time, coinciding with the development of light chain escape and disease progression. This emphasises the power of cfDNA from both diagnostic and monitoring perspectives in advanced MM.

These findings were validated in a separate study that examined the clonally rearranged IgH gene in MM utilising an NGS technique, in contrast to a targeted panel of known recurrent mutations^[41]^. This study found that the clonal IgH rearrangements in cfDNA became undetectable in patients who responded to therapy before a change in the paraprotein, suggesting cfDNA is a more immediate estimation of tumour burden than the traditional methods of monitoring. This approach is limited by the issues with IgH analysis mentioned above but validates the ability of cfDNA to monitor response in MM patients.

Finally, cfDNA analysis in MM can be correlated with survival. In one study of MM patients, paired mutational analysis of both bone marrow DNA and plasma ctDNA in both newly diagnosed and relapsed refractory cases were examined^[42]^. Patients with more than two mutations, or a fractional abundance of a mutation greater than 1%, had significantly shorter overall survival. These findings were validated by another study, where a higher absolute number of mutations, as well as a higher fractional abundance of these mutations, was also associated with worse overall survival^[38]^.

### cfDNA in lymphomas

There is literature surrounding cfDNA in a number of Hodgkin’s and NHL, but arguably the best developed area of haematology is in diffuse large B-cell lymphoma (DLBCL). DLBCL is the most common subtype of NHL. In the field of DLBCL, cfDNA has been demonstrated to be useful in a number of areas, including diagnosis, prognosis and treatment. For a number of reasons, DLBCL as a disease stands to benefit from the use of cfDNA technology. The current standard for monitoring DLBCL response to therapy is PET/CT scans or other imaging techniques^[43]^, with associated radiation risks and a limit of detection that may miss residual disease. DLBCL diagnosis is based on morphology and the immunophenotype of tumour cells, but there is increasing use of molecular testing, for example to identify cell-of-origin or other targetable mutations that provide useful prognostic information^[43]^. This emerging need for molecular analysis may benefit from increasing use of cfDNA. Studies have used cfDNA in the diagnosis of DLBCL and analysis of the tumour genome, as well as in prognostication and monitoring of patients in remission.

There is promise for cfDNA in the diagnosis in some DLBCL subtypes. Current diagnostic criteria based on morphology and immunophenotype may be problematic in situations where a suitable tissue sample is difficult to obtain but cfDNA identified in body fluids may allow the identification of important diagnostic information. An excellent example is primary central nervous system lymphoma, an aggressive subtype of DLBCL that is difficult to diagnose because of the inherent risks of central nervous system (CNS) tissue biopsies. cfDNA has been identified in the cerebrospinal fluid of patients with CNS tumours^[44,45]^. The identification of lymphoma-associated mutations or clonal IgH rearrangements within the cerebrospinal fluid may provide a surrogate diagnostic criterion in these cases, although further studies are required to confirm the utility of this approach before it enters clinical use^[46]^.

cfDNA has also been used for the genotyping of DLBCL. In one study, NGS-based techniques were used to correlate the presence of lymphoma-associated genes in the cfDNA and tumour biopsies^[34]^. This study identified mutations in the blood that were the same as the diagnostic tissue biopsy with a concordance rate of 85%. Further studies have used ancer Personalised Profiling by deep sequencing, a technique validated and FDA-approved in non-small cell lung cancer, to interrogate ctDNA in DLBCL^[47-49]^. Rossi *et al*.^[49]^ demonstrated that mutations with an allele frequency of > 20% in the primary tumour sample were reliably detectable in the cfDNA compartment.

Importantly, tumour genotyping by cfDNA does not simply mirror the genotype obtained from tumour tissue biopsies; it is complementary. Spatial and temporal heterogeneity of haematological neoplasms is increasingly recognised^[50]^, and cfDNA interrogation provides a representation of the whole-tumour genome rather than that of a single biopsy site. These mutations, identified in cfDNA but not in original tumour tissue biopsies, have been confirmed to be real in a number of confirmatory studies^[48,49,51]^.

Specific mutations, when identified in DLBCL, are known to predict response to treatment. For example, MYD88 mutations may correlate with response to ibrutinib^[52]^. Early studies have shown that current techniques can identify such mutations in cfDNA^[53]^. Prospective studies are required before cfDNA is used to guide treatment choices, but the increasing use of personalised medicine in other fields indicates the potential value of such a technique.

In DLBCL, cfDNA can also provide prognostic information. Using IgH sequencing, Kurtz *et al*.^[30]^ showed that quantification of ctDNA in DLBCL patients correlates closely with metabolic tumour volume. Scherer *et al*.^[48]^ identified a prognostic correlation using an NGS-based technique, showing ctDNA is a strong predictive factor at diagnosis, with higher levels of ctDNA correlating with inferior progression free survival and a higher risk of disease progression. Interestingly, in the pre-transplant setting, a higher ctDNA level prior to allogeneic stem cell transplant for lymphoma was not prognostic of outcome, but the change in ctDNA levels after transplant correlated with response and predicted relapse^[54]^. Aberrant methylation of cfDNA has also been identified in DLBCL patients and has been correlated with survival^[55,56]^. In one study, the methylation of key lymphoma-associated genes detectable in the cfDNA was assessed, finding that aberrant methylation of the *DAPK1* gene was associated with worse five-year survival^[57]^. This study was validated in part by a further study that identified global hypomethylation of DNA in both tumour and blood samples of DLBCL patients, showing global hypomethylation was associated with aberrant DAPK1 promoter methylation and shared the same poor prognostic impact^[58]^.

One of the more exciting implications of cfDNA in DLBCL is that it can be used to assess response to therapy and monitor for relapse. In a retrospective study, patients who subsequently progressed were identified; those who had persistent ctDNA in the plasma had the shortest time to progression^[59]^. In patients treated for DLBCL, ctDNA became undetectable in patients who responded to therapy, while it remained detectable in those patients who were resistant, frequently with the emergence of new mutations^[49]^. Response can be predicted by the change in ctDNA levels between Days 0 and 15 of therapy, with a failure to decrease levels at Day 15 predicting a lack of response with high specificity in one study^[60]^. In some lymphomas, interim PET scans are used to escalate or reduce chemotherapy based on response to initial cycles of chemotherapy; this is not yet used in DLBCL. While the above studies of ctDNA show that there is a correlation between interim ctDNA measurement and outcome, there are no studies that show altering treatment based on this result will improve a patient’s outcome and further studies are required to confirm this prior to integration into clinical practice.

Other lymphomas with cfDNA data include classic Hodgkin’s lymphoma, where detection of specific mutations in plasma correlated with tissue biopsies and may be of prognostic significance but confirmatory studies are pending^[61]^. Intravascular large B-cell lymphoma, a notoriously difficult diagnosis to make due to its intravascular location, has also been investigated with targeted sequencing and digital PCR of cfDNA, identifying B-cell associated mutations in 88% of diagnostic samples in one small study^[62]^. There is minimal literature about cfDNA in T-cell lymphomas, likely due to the rarity of these conditions, although one paediatric study found lower proportions of ctDNA in anaplastic large cell lymphoma when compared to B-cell or Hodgkin’s lymphomas^[63]^. In angioimmunoblastic T-cell lymphoma, another challenging diagnosis typically requiring multiple biopsies, one study showed that interrogation of cfDNA can detect recurrent mutations and that these mutations became undetectable in patients who responded to therapy^[64]^.

### Myeloid disorders

There is far less literature about the role of cfDNA in myeloid disorders, such as myeloproliferative disorders, myelodysplastic syndromes (MDS) and acute myeloid leukaemias (AML). In MDS and AML, the ease of access to circulating disease would intuitively suggest a reduced benefit of cfDNA compared to tumour-based DNA assessments. Interestingly, this is not the case. One study examined 24 patients with AML comparing BM and cfDNA samples, finding that cfDNA identified more mutations than found in the BM^[50]^ with a median of three cfDNA mutations in 83% of patients that were not detectable in the BM sample. Furthermore, a significant number of patients had potentially actionable mutations found in cfDNA that were not seen in the BM samples. This highlights once again the marked spatial genetic heterogeneity of haematological malignancies, including those with circulating disease.

In AML and MDS patients after allogeneic stem cell transplant, the monitoring of ctDNA can be used to predict relapse and the presence of ctDNA at one month and three months post-transplant correlates with survival^[65,66]^. This may identify patients in whom early interventions, such as weaning of immunosuppression, may reduce relapse rates. Furthermore, ctDNA can be used for chimerism studies to analyse engraftment kinetics^[65]^.

The myelodysplastic syndromes are another area of haematology where cfDNA may prove highly beneficial in filling an unmet need. Current diagnostic criteria rely on morphology and cytogenetic abnormalities; despite the application of strict criteria, the interpretation of morphology remains subjective, and cytogenetic abnormalities are useful but are only found in 50% of cases^[67]^. Furthermore, genetic information is often compromised by hypocellularity of bone marrow samples. Molecular techniques have identified recurrent somatic mutations in MDS, although these are not yet included in diagnostic criteria. cfDNA may therefore represent a useful, easily accessible source of genetic material for recurrent sampling in MDS patients.

In a study of MDS patients on a clinical trial, serial bone marrow and cfDNA samples were analysed; 10/12 patients had driver mutations identified on the BM through next-generation sequencing^[68]^. Digital PCR was performed to validate these results; importantly, the variant allele fractions correlated well between the two sites, and this occurred even when there were marked peripheral blood cytopenias. Importantly, there was no correlation between cfDNA and the white blood cell count in peripheral blood, which suggests the source of cfDNA, while haemopoietic, is the BM rather than peripheral blood cells^[68]^. This study also identified clonal evolution in several patients, including the emergence of mutations associated with transformation to acute leukaemia, and the ctDNA levels changed reflecting tumour burden.

### Other haematological conditions

Another disease in which cfDNA may play a key role in the future is aplastic anaemia (AA). AA is an autoimmune condition where T-cell mediated destruction of haemopoietic stem cells leads to a markedly hypocellular bone marrow and peripheral blood cytopenias; the hypocellularity of the marrow renders traditional techniques to analyse the underlying genetics insensitive^[69]^. In practice, AA is frequently associated with clonal disorders of haemopoiesis, including MDS and paroxysmal nocturnal haemoglobinuria. A study of AA patients identified that mutations were identified more frequently in cfDNA than in BM samples, with 18% of patients having detectable mutations in cfDNA but not the BM^[69]^. Thus, cfDNA may be useful in AA patients, but also displays a proof of concept in patients with hypocellular bone marrow that historically makes genetic assessment challenging.

## Conclusion and future directions

Interrogation of cfDNA via liquid biopsy holds promise in a range of haematological conditions. cfDNA provides genetic information at diagnosis that can be missed by other biopsy techniques, providing complementary information to current gold standard diagnostic techniques. Changes in cfDNA over time correlate with survival and response to therapy, and changes in cfDNA can predict disease progression.

These findings open the door for cfDNA to guide therapeutic decisions in the clinic, for example through the identification of targetable lesions at diagnosis as well as by changing therapies on the basis of early prediction of poor response. There is less literature surrounding cfDNA in myeloid conditions; however, the ability of cfDNA analysis to identify genetic abnormalities in many myeloid conditions is undisputable.

The next step for cfDNA in haematology is incorporation into clinical trials to better characterise the clinical utility of these findings. Some questions to answer are challenging: What should be done when a patient is in a minimal residual disease negative remission by traditional minimal residual disease assessments, but is positive by cfDNA? What is the optimal therapy for a patient who has a targetable mutation in the cfDNA that is not identified on the traditional biopsy? There is indisputable promise in cfDNA for the haematology patient, but such questions need to be addressed in clinical trials before cfDNA can be confidently applied for haematology patients in the clinic.

## Declarations

### Copyright

© The Author(s) 2020.
